# Distal Landing Zone-Related Complications of Conventional Endovascular Aneurysm Repair (EVAR) in the Long Term: A Comprehensive Systematic Review

**DOI:** 10.7759/cureus.77379

**Published:** 2025-01-13

**Authors:** Apostolos G Pitoulias, Charalampos N Loutradis, Dimitrios A Chatzelas, Matthaios G Pitoulias, Loukia A Politi, Vangelis Bontinis, Georgios Pitoulias

**Affiliations:** 1 Division of Vascular Surgery, Second Department of Surgery, "G. Gennimatas" General Hospital, Aristotle University of Thessaloniki, Thessaloniki, GRC; 2 Department of Vascular Surgery, AHEPA University General Hospital, Aristotle University of Thessaloniki, Thessaloniki, GRC

**Keywords:** cranial limb migration, distal landing zone, endoleak type ib, endovascular aneurysm repair, evar, follow-up

## Abstract

The aim of this systematic review is to evaluate the late-onset complications considering the distal landing zones (DLZ) in patients with abdominal aortic aneurysm (AAA) treated by conventional endovascular aneurysm repair (EVAR). We conducted a systematic review of electronic databases, clinical trial registries, and grey literature to retrieve studies on this issue. The inclusion criteria could be summarized as follows: (1) observational studies or case series with more than 30 patients included, (2) at least three-year follow-up, and (3) endpoints including endoleak type Ib (ELIB) or cranial iliac limb migration/retraction (CLR) or DLZ failure-related reinterventions or other complications. Of the 517 potentially eligible studies, the detailed search revealed eight articles with a total of 2569 patients for further investigation. Due to the extreme heterogeneity in definitions and reporting outcomes among the included studies, the synthesis and meta-analysis of data were not plausible. A quantitative assessment of reported outcomes revealed a pooled incidence of 2.1% for ELIB, 1.7% for CLR, and 5.7% for DLZ failure-related reinterventions. Data were considered controversial to extract a consensus for the dilatation of the DLZ. This systematic review delineates the importance of late-onset complications originating from DLZ failures for the longevity of an EVAR procedure and gathers the current knowledge regarding the magnitude and clinical implications of DLZ failure from the existing literature and in the best available quality. Current literature data show a blurred image regarding the long-term morphological alterations of iliac arteries and especially in the impact of DLZ dilatation and emphasize the necessity of prolonged follow-up for at least five years.

## Introduction and background

Introduction

The prevalence of abdominal aortic aneurysm (AAA) has been reported to vary between 0.5% and 6.7% in different countries worldwide with an overall pooled incidence of 4.8% [1]. The degenerative nature of underlying pathophysiological mechanisms and the continuous affection of the abdominal arterial wall by predisposing factors such as the advancement of age, hypertension, smoking, etc. challenge the long-term outcomes of surgical treatment and especially of endovascular aneurysm repair (EVAR) [1,2]. As the population continues to age, late-onset complications arising after an EVAR procedure tend to become increasingly common.

The superiority of EVAR over open surgical repair for the 30-day operative mortality and graft-related complications and reinterventions has been highlighted early in the literature by the DREAM trial [3]. However, the early benefits of EVAR were questioned from the outcomes of the United Kingdom EVAR trial, which showed increased rates of EVAR-related complications and reinterventions in the long term [4]. The maintenance of the superior early outcomes of EVAR in the long term seems to be dependent on the early recognition and avoidance of two major EVAR pitfalls: type I endoleaks and stent graft migrations [5]. However, despite the continuous evolvement and improvement of the available EVAR stent grafts, Li et al. showed that EVAR is still associated with higher long-term reintervention and secondary rupture rates compared to open repair and this has significant implications for the overall final cost of the endovascular technique in the long term [6].

The late-onset complications are attributed to the steadily but surely post-EVAR remodeling of the aorta and aortoiliac bifurcation [7-9]. A major component of a successful and long-lasting EVAR procedure is the maintenance of the seal at the proximal and distal landing zones (PLZ and DLZ) [10]. To date, several previous studies and meta-analyses have thoroughly evaluated the characteristics and potentially preventive factors of PLZ failures and highlighted the importance of proper recognition and treatment of type Ia endoleak [10-12]. In contrast, only a few small studies and post-hoc analyses have evaluated the characteristics of the endoleak type Ib (ELIB) and the long-term complications, which are related to the loss of sealing due to DLZ failure [13-20]. Thus, the factors leading to iliac remodeling after EVAR and its association with possible graft failures are not yet well documented. 

The aim of this systematic review is to explore and analyze the epidemiology and clinical significance of DLZ failures and to evaluate its effects on late-onset complications in AAA patients treated with conventional EVAR.

Methods

The Preferred Reporting Items for Systematic Reviews and Meta-Analyses (PRISMA) checklist was implemented for this review [21].

Data Source and Search Strategy

We included studies evaluating the long-term outcomes of the DLZ sealing in patients with AAA. The inclusion criteria were defined as follows: (1) observational studies, (2) studies or case series with more than 30 patients included, (3) studies with a postoperative follow-up of at least three years, and (4) studies with endpoints including ELIB, and/or distal migration, and/or limb retraction, and/or cranial limb migration, and/or other complications affecting the DLZ. Since our study concerns the DLZ results of conventional EVAR, we excluded studies reporting on the performance of iliac branch devices and studies with more advanced and complicated EVAR techniques, such as chimney and fenestrated EVAR, to avoid any possible spurious influence on our results by complications due to other reasons other than the DLZ failure.

A systematic search of the literature in PubMed, Google Scholar, and the Cochrane Central Register of Controlled Trials (CENTRAL) was performed (January 2021 until June 2023). The following terms were used: ((EVAR) OR (endovascular aneurysm repair)) AND (abdominal aorta) AND ((post EVAR complications) OR (iliac aneurysm) OR (endoleak type Ib) OR (migration) OR (distal landing zone failure). Also, a manual search using the terms EVAR, iliac, migration, cranial, postoperative, limb, distal, and aneurysm in various combinations was also performed. After the extraction of the results of the search, duplicated articles were excluded, whereas, in the case of metachronous publications from the same institution, only the latest article or the article with the largest number of patients was included. The study selection was continued with exclusion by title, by abstract, and finally by text. We also searched manually the references of the relevant studies, the abstracts of the relevant congresses, and the ClinicalTrials.gov registry to identify ongoing and completed registered trials. 

Study Selection and Data Extraction

The search results were evaluated by two independent reviewers (AGP and DAC). Any disagreement that occurred was evaluated by a third independent reviewer (GAP). The quality of the included manuscripts was evaluated using the Newcastle-Ottawa Scale (NOS) for assessing the quality of nonrandomized studies in meta-analyses [22]. Using this scale, the manuscripts were awarded stars based on their selection of the study groups, the comparability of the groups, and the exposure or outcome of interest. Every manuscript that received more than 6 stars was defined as a high-quality study for our systematic review [22].

Data Analysis

We evaluated the possibility of quantitative synthesis by using a data extraction form for all studies designed according to the Cochrane Checklist of Items, containing fields for all important data on trial design, demographics, outcome measurements, and details relevant to the assessment of the risk of bias. Each study form was again completed by the two independent reviewers (AGP and DAC), and disagreements on study selection and data collection were evaluated by a third independent reviewer (GAP).

A significant heterogeneity among studies was evident with regard to the definition used for the DLZ failure and the affecting factors which can be summarized as follows: absence of preoperative common iliac artery diameter (CID) anatomical and operative data, absence of a common definition for reporting DLZ failure based in the postoperative CID, and other anatomical characteristics, i.e., aortoiliac or iliac angulations, etc. In particular, the definition of reported postoperative CID as a predisposing factor for DLZ failure was different in all studies and ranged from >10 to >21 mm. Moreover, significant differences among the studies were noted regarding the population included (individual limbs or patients, reports in specific age or sex cohorts, cases with aneurysmal iliac arteries) and the methodology followed (i.e., mainly post-hoc analyses of secondary outcomes with different primary objectives). On this basis, all reviewers consented that a reliable quantitative synthesis and a meta-analysis of data were not feasible within the frame of this systematic review, which inevitably was limited to gathering the current knowledge regarding the magnitude and clinical implications of DLZ failure from the existing literature and in the best available quality. In contrast, we only performed a quantitative assessment of the pooled incidence for the studied outcomes to present an overall summary of the included studies.

## Review

Results

Search Results

**Figure 1:**
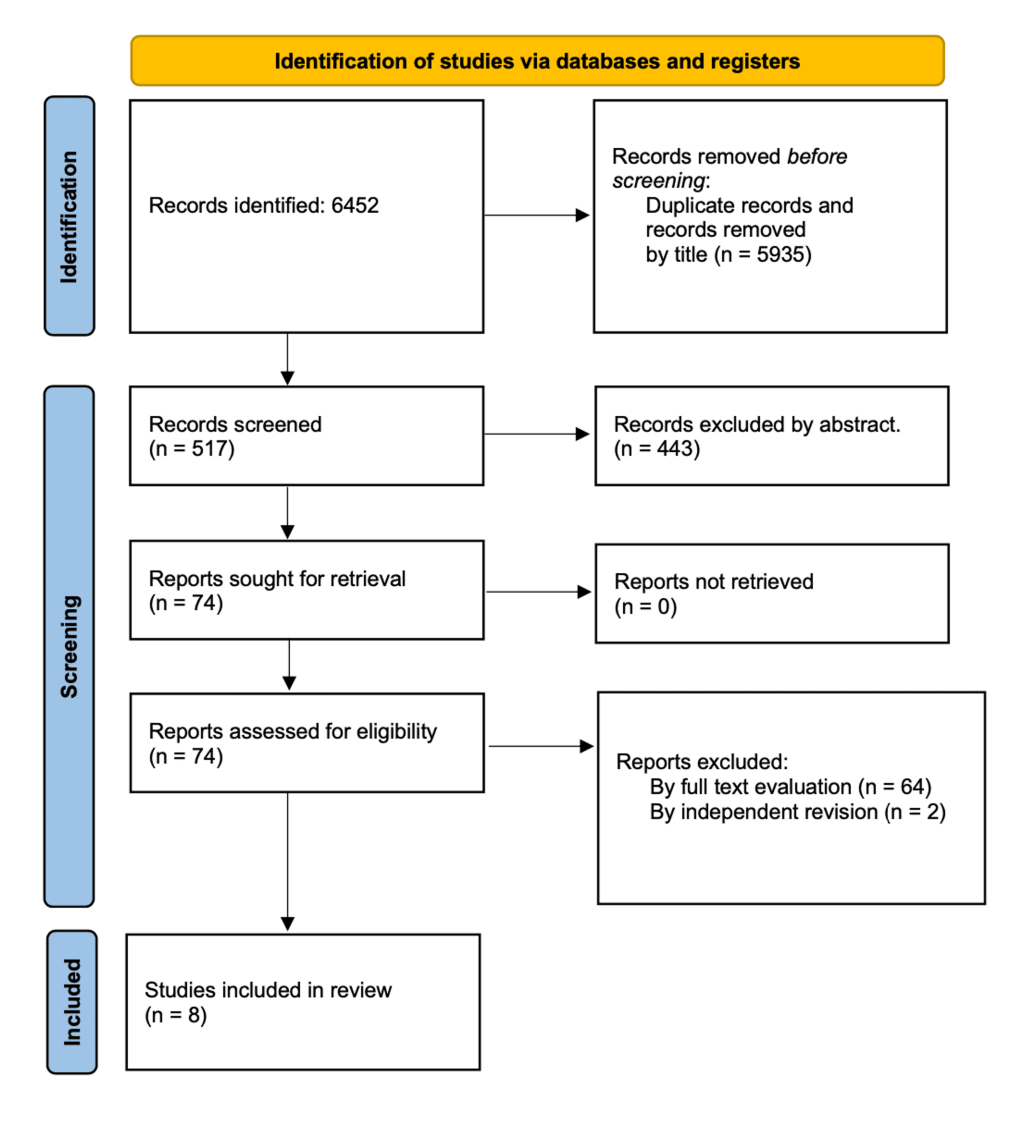
Flow diagram of search results

Figure 1 depicts the flow diagram of the studies included. During the initial search, a total of 6452 articles were identified from the combined search of PubMed, Google Scholar, the Cochrane Central Register of Controlled Trials (CENTRAL), ClinicalTrials.gov, and abstract archives. After the exclusion of duplicated manuscripts and exclusion by title, 517 articles were further processed. A total of 443 records were deemed non-eligible following the evaluation of the abstract. Of the remaining 74 records, 64 of them were excluded after the evaluation of the full text. Two of the remaining 10 manuscripts were excluded after independent revision due to limitations in methodology and/or lack in data presentation [23,24]. Finally, eight articles remained for further analysis [13-20].

Study Quality

Table 1 summarizes the qualitative assessment and the demographic characteristics of the included studies. All studies were assessed as moderate quality: three studies had NOS 5, four had NOS 6, and one had NOS 7. In particular, the studies from Barry et al. [14] (1 point for selection, 1 for comparability, and 3 for outcome), from Janho et al. [15] (2 points for selection, 0 for comparability, and 3 for outcome), and from Telles et al. [16] (1 point for selection, 1 for comparability, and 3 for outcome) received a total of 5 stars. Those from Roos et al. [13] (3 points for selection, 1 for comparability, and 2 for outcome), Sirignano et al. [17] (3 points for selection, 1 for comparability, and 3 for outcome), Bastos Gonçalves et al. [19] (2 points for selection, 1 for comparability, and 3 for outcome), Mascoli et al. [18] (3 points for selection, 1 for comparability, and 2 for outcome), and Griffin et al. [20] (2 points for selection, 1 for comparability, and 3 for outcome) were considered as higher-quality studies. 

**Table 1:** Qualitative assessment and demographic characteristics of the included articles CID* (mm) is used by authors as a cutoff point for subgroup analysis. Age and follow-up duration are in mean±SD or median and interquartile range. NOS: Newcastle-Ottawa Scale; CID: common iliac artery diameter

Study, date	NOS	N	CID* (mm)	Age (years)	Sex (male)	Follow-up (months)
Roos et al., 2017 [13]	6	444	>18	74.9±7.4	376 (84.7)	46 (2-92)
Barry et al., 2021 [14]	5	711	>9.6	75.7±7.1	0 (0)	60 (NA)
Janho et al., 2019 [15]	5	288	>21	70 (31)	224 (77.7)	60.0±NA
Telles et al., 2016 [16]	5	38	>21	70.4±8.2	34 (89.5)	25.8±14.9
Sirignano et al., 2016 [17]	7	49	>12.7	57.4±2.7	47 (96)	56.8±42.7
Mascoli et al., 2019 [18]	6	616	>15	73.0±9.0	15 (34)	60.4 (41)
Bastos Gonçalves et al., 2017 [19]	6	341	>14	72.3±7.5	300 (88)	52.8 (31.2)
Griffin et al., 2015 [20]	6	82	>20	72.0±8.0	76 (92.6)	49.3 (25)

Study Outcomes

The quantitative assessment of the studies included in this systematic review is presented in Table 2. The stent graft devices used for the EVAR procedure in the included studies are presented, in detail, in Table 3. All included studies were retrospective analyses of prospectively collected data and reported adequately on the incidence of ELIB as well as of DLZ failure-related rupture and reintervention rates. Seven studies [13-18,20] reported these results in patients, while two of them [16,20] reported the outcomes of DLZ dilatation in iliac arteries instead of patients. Additionally, in one study [19], all outcomes are reported in iliac arteries. Four studies reported outcomes in special cohorts of populations: Barry et al. [14] reported outcomes in a large cohort of female patients, Sirignano et al. [17] in patients younger than 60 years old, and Telles et al. [16] and Griffin et al. [20] in cohorts of EVAR patients with aneurysmatic iliac arteries. However, the total of eight included studies summarizes the outcomes in a considerable number of 2569 patients. The pooled incidences of ELIB and DLZ failure-related reinterventions in seven studies reporting a total of 2228 patients were 2.1% and 5.7%, respectively. Similarly, the respective rates in the single study reporting in iliac arteries were 2.7% and 5.7%. Furthermore, a high degree of agreement was observed between studies in DLZ failure-related rupture which was 0.2% in the seven series reporting in patients and 0.2% in the single study reporting in iliac arteries.

**Table 2:** Quantitative assessment of the included articles *: Limb cranial migration/retraction: the pooled incidence in seven studies and 2228 patients is 0.6%, and it is 1.7% if only the three studies (814 patients) reporting on migration are taken into account. #: DLZ failure related. ^: Bastos Gonçalves et al. reported outcomes in 341 patients and 597 distal iliac zones. &: Telles et al. reported outcomes in 38 patients and DLZ dilatation in 76 iliac arteries. ": Percentage in 837 iliac arteries. Iliac limb occlusions were reported to be 3% in 711 patients as follows: Griffin et al. 4.9% (n=4), Janho et al. 2.4% (n=7), and Bastos Gonçalves et al. 2.9% (n=10). ELIB: endoleak type Ib

Study, date	N	ELIB, n (%)	Migration*, n (%)	Dilatation, n (%)	Rupture^#^, n (%)	Reintervention^#^, n (%)
Studies reporting outcomes in patients						
Roos et al., 2017 [13]	444	10 (2.3)	10 (2.3)	9 (2)	0 (0)	24 (5.4)
Barry et al., 2021 [14]	711	9 (1.3)	0 (0)	0 (0)	1 (0.1)	59 (8.3)
Janho et al., 2019 [15]	288	6 (2.1)	3 (1)	0 (0)	1 (0.3)	13 (4.5)^"^
Telles et al., 2016 [16]^&^	38	4 (10.5)	0 (0)	-	0 (0)	7 (18.4)
Sirignano et al., 2016 [17]	49	3 (6.1)	0 (0)	0 (0)	0 (0)	3 (6.1)
Mascoli et al., 2019 [18]	616	14 (2.3)	0 (0)	0 (0)	2 (0.3)	14 (2.3)
Griffin et al., 2015 [20]	82	0 (0)	1 (1.2)	-	0 (0)	6 (7.3)^"^
Overall, in patients	2228	46 (2.1)	14 (0.6-1.7)*	NA	4 (0.2)	126 (5.7)
Studies reporting outcomes in iliac arteries						
Telles et al., 2016 [16]^&^	76	-	-	18 (23.7)	-	-
Bastos Gonçalves et al., 2017 [19]^	597	16 (2.7)	54 (9)	295 (49.4)	1 (0.2)	34 (5.7)^"^
Griffin et al., 2015 [20]	164	-	-	146 (89.2)	-	-
Overall, in iliac arteries	837	16 (2.7)	54 (9)	459 (54.8^»^)	1 (0.2)	34 (5.7)

**Table 3:** Used stent graft devices The table showcases the number of used stent graft devices in each study.

Study, date	Talent	Excluder	Treovance	Zenith	Other (Apollo, AFX, Nellix, Ovation, Incraft)	Endurant
Roos et al., 2017 [13]	16/444 (3.6%)	53/444 (12%)	-	78/444 (17.7%)	5/444 (1.1%)	292/444 (66.3%)
Barry et al., 2021 [14]	-	538/711 (75.6%)	-	40/711 (5.6%)	-	133/711 (18.7%)
Janho et al., 2019 [15]	52/288 (18%)	3/288 (1%)	3/288 (1%)	11/288 (3.8%)	-	219/288 (76%)
Telles et al., 2016 [16]	25/38 (66%)	-	-	7/38 (18%)	6/38 (16%)	-
Sirignano et al., 2016 [17]	11/49 (22.5%)	17/49 (34.7%)	1/49 (2%)	2/49 (4%)	8/49 (16%)	10/49 (20%)
Mascoli et al., 2019 [18]	-	-	-	-	-	-
Bastos Gonçalves et al., 2017 [19]	22/599 (3.6%)	238/599 (39.7%)	-	5/599 (0.83%)	12/599 (2%)	322/599 (53.7%)
Griffin et al., 2015 [20]	10/120 (8.3%)	16/120 (13.3%)	-	94/120 (78.3%)	-	-

On the contrary, significant differences were observed among included studies in cranial iliac limb migration/retraction (CLR) and DLZ dilatation rates. Particularly, in four studies [14,16-18], a complete absence in reporting on CLR was noted, and in four studies [14,15,17,18], post-EVAR dilatation of iliac arteries at DLZ was not reported, which allows for the assumption that the authors of these studies probably did not include the investigation for these aspects of outcomes in their research interest at all. Focusing on the data of the remaining studies, we recorded the following. The pooled incidence of CLR in 814 patients reporting on migration in patients was 1.7% (n=14), while Bastos Gonçalves et al. [19] in 597 iliac limbs observed 54 (9%) cases of migration. Regarding the DLZ dilatation, the single-center study by Roos et al. [13] with 444 conventional EVAR patients noted that dilatation occurred in nine cases (2%). On the other hand, studies reporting in iliac limbs [16,19,20] showed a much greater incidence of DLZ dilatation. The single-center study by Griffin et al. [20] compared the outcomes related to the DLZ after EVAR between 57 patients with aneurysmatic DLZ (mean age 72±8 years, 70 common iliac arteries with a diameter of ≥20 mm) and 25 controls with normal DLZ (mean age 73±7 years, 50 iliac arteries with a diameter of ≤15 mm) and reported 89.2% incidence of DLZ dilatation over a median follow-up of 39.2 and 49.3 months, respectively. Results showed a greater CID change in the aneurysmal DLZ compared to the normal DLZ group (0.09 mm/m, 95% CI 0.07-0.1 vs 0.03 mm/m, 95% CI -0.009-0.07; p<0.0001). One additional retrospective small study from Telles et al. [16] evaluated the DLZ outcomes after EVAR in 38 patients (mean age 70.4±8.2 years), which were followed up for 25.8±14.9 months, and the dilation of the common iliac artery was evident in 18 of 76 iliac limbs with an incidence of 23.7%. Finally, the large retrospective study by Bastos Gonçalves et al. [19] evaluated the outcomes of DLZ after EVAR in 341 patients (mean age 72.3±7.5 years) and reported outcomes in 597 common iliac arteries, over a median follow-up of 4.7 years. The authors found that CID dilatation ≥20% occurred in 49.4% of iliac arteries and it was ≥4 mm in 39.3%. 

The pooled incidence of iliac limb occlusions in three studies reporting in a subtotal of 711 patients was 3%. Table 2 summarizes the results of all aspects of DLZ failures and provides the available data on iliac limb occlusions.

Discussion

The long-term success of an EVAR procedure depends largely on the maintenance of the sealing between the endoprosthesis and vessel wall at the level of the proximal neck and DLZ [7]. Although the postoperative dilatation of the proximal neck and its impact on the maintenance of sealing at the PLZ has been thoroughly investigated [5,11,12], factors such as the progressive angulation of the aortoiliac segment and the ongoing aneurysmatic dilatation of the DLZ after EVAR as well as their long-term clinical significance have so far not been systematically researched. To this end, current evidence suggest that the postoperative arterial wall remodeling of the proximal neck and the iliac arteries is a major determinant of successful long-term sealing [25,26]. 

In this systematic review, we aimed to evaluate and analyze the long-term DLZ-related complications of EVAR, including relevant studies with long-term follow-up. Contrary to the plethora of articles reporting on the long-term data of proximal aortic neck-related failures of EVAR [5-9,11,12], reliable data on DLZ failures are occasionally reported, and it seems that this issue has not received the appropriate attention, in relation to the severity of its potential complications. Inevitably and despite our extensive systematic review of the literature, we could only identify eight eligible studies meeting the inclusion/exclusion criteria as well as the quality standards for a proper systematic review. All included studies were retrospective observational analyses of prospectively collected data [13-20]. Importantly, in the lack of universal definitions in relevant guidelines, all included studies used vague anatomical definitions and features as well as heterogeneity in definitions of reported outcomes and endpoints. Thus, a quantitative synthesis and meta-analysis of results are not realistically feasible and reliable, leaving the accomplishment of a systematic review as the only option for drawing conclusions on the issue of long-term EVAR complications due to DLZ failures.

Currently, the relevant evidence suggested that the long-term rate of ELIB is about 2.1%, ranging in different studies from 0% to 10.5%. The CLR, which might be also a causative factor of ELIB, was documented in four studies only. In three studies [13,15,20], the pooled incidence of migrations was 1.7%, ranging from 1% to 2.3% with Bastos Gonçalves et al. [19] documenting a rate of 9%, while four studies did not mention migrations at all [14,16-18]. These wide differences in outcomes reflect the absence in definitions of endpoints and in universal reporting standards. The incidence of limb occlusions and late-onset ruptures were rare with rates of 3% and 0.2%, respectively. All included studies reposted reintervention rates with a pooled incidence of 5.7%, ranging from 2.3% in cohorts of the general post-EVAR population [18] to as high as 18.4% in cohorts of patients with aneurysmatic iliac arteries [17] treated by conventional EVAR.

The included studies revealed a notable variation in rates of DLZ dilatation, ranging from 0% to over 20%. Interestingly, four [14,15,17,18] out of the eight studies did not include any results on postoperative dilatation, while one out of eight documented rates of just 2% [13]. On the other hand, in two manuscripts by Telles et al. [16] and Griffin et al. [20], the authors documented extreme dilatation of the iliac arteries with impending loss of seal, ranging from 23.7% to 89.2%, respectively, without specifying the percentage of dilatation. Bastos Gonçalves et al. [19] in their work documented 295 cases of iliac dilatation >20% with impending loss of seal in 170 cases. This issue derives from the different definitions used for dilatations across the included studies, as some studies defined dilatation in relative percentages while others in absolute values. The lack of a commonly accepted definition in relevant guidelines may have resulted in this significant heterogeneity demonstrated among the included studies but, more importantly, in the lack of a true estimation on the actual rates of DLZ dilatation. 

Although the pathophysiological factors for the post-EVAR remodeling of the PLZ and DLZ are not well investigated, some authors suggest that they should be the same as the ones leading to the expansion of the aorta and the development of AAA in the first place [27]. AAA is a degenerative disease that can be characterized by failure of the structural proteins of the aortic wall. The decrease of elastin and collagen which is apparent in AAA can lead to the gradual weakening of the aortic wall and therefore can result in a more pliable and prone-to-deformation aorta [28]. Furthermore, Bruijn et al. [29] performed a study with the histological evaluation of post-EVAR aorta samples and showed that hypertrophic scarring and decreased inflammatory response were apparent in the aortic wall after EVAR, indicating a hypoxic response after the implantation of the endoprosthesis. The combination of these well-known pathophysiological findings results in an aorta with pliable and prone-to-deformation segments, while other segments are fibrotic and unresponsive to changes. This mismatch in flexibility and pliability between these segments creates an environment, which in combination with the hemodynamic changes in shear stress and pressure distribution among the aortic wall can lead to the development of post-EVAR remodeling and might result in long-term DLZ failure.

Acknowledging the limitations of the current systematic review, with the inclusion of retrospective observational studies with potential selection bias and principally with the lack of a single homogeneous protocol of reporting outcomes, we believe that clarified the incidence of long-term incidence of ELIB which appears to be at the level of 2.1%, as well as the incidence of clinically severe CLR and DLZ failure-related reinterventions which varied at approximately 1.7% and 5.7%, respectively. However, factors like the progressive angulations of the aortoiliac segment through time and the ongoing aneurysmatic dilatation of the DLZ after EVAR remain not well defined and require further clinical investigation.

## Conclusions

With an aging population, late-onset complications related to DLZ failures are becoming more common, jeopardizing the procedure's longevity and necessitating reinterventions or posing life-threatening risks. Although morphological changes at the DLZ occur, their clinical significance remains unclear. Therefore, multicenter randomized studies with clear endpoints and long-term follow-up protocols focusing on the DLZ are needed to identify risk factors and minimize reinterventions.
